# Network pharmacology and molecular docking-based analysis of protective mechanism of MLIF in ischemic stroke

**DOI:** 10.3389/fcvm.2022.1071533

**Published:** 2022-11-18

**Authors:** Mengting Lv, Qiuzhen Zhu, Xinyu Li, Shanshan Deng, Yuchen Guo, Junqing Mao, Yuefan Zhang

**Affiliations:** ^1^School of Medicine, Shanghai University, Shanghai, China; ^2^The Seventh People's Hospital of Shanghai University of Traditional Chinese Medicine, Shanghai, China; ^3^College of Pharmacology, Anhui University of Chinese Medicine, Hefei, China; ^4^Department of Clinical Pharmacy, Jiading Branch of Shanghai General Hospital, Shanghai Jiao Tong University School of Medicine, Shanghai, China

**Keywords:** monocyte locomotion inhibitory factor (MLIF), ischemic stroke (IS), network pharmacology, molecular docking, JNK

## Abstract

**Objective:**

This study aimed to evaluate the potential mechanism by which Monocyte locomotion inhibitory factor (MLIF) improves the outcome of ischemic stroke (IS) inflammatory injury.

**Methods:**

Potential MLIF-related targets were predicted using Swiss TargetPrediction and PharmMapper, while IS-related targets were found from GeneCards, PharmGKB, and Therapeutic Target Database (TTD). After obtaining the intersection from these two datasets, the Search Tool for Retrieval of Interacting Genes/Protein (STRING11.0) database was used to analyze the protein-protein interaction (PPI) network of the intersection and candidate genes for MLIF treatment of IS. The candidate genes were imported into the Metascape database for Gene Ontology (GO) functional analysis and Kyoto Encyclopedia of Genes and Genomes (KEGG) pathway enrichment. The top 20 core genes and the “MLIF-target-pathway” network were mapped using the Cytoscape3.9.1. Using AutoDock Vina1.1.2, the molecular docking validation of the hub targets and MLIF was carried out. In the experimental part, transient middle cerebral artery occlusion (tMCAO) and oxygen and glucose deprivation (OGD) models were used to evaluate the protective efficacy of MLIF and the expression of inflammatory cytokines and the putative targets.

**Results:**

MLIF was expected to have an effect on 370 targets. When these targets were intersected with 1,289 targets for ischemic stroke, 119 candidate therapeutic targets were found. The key enriched pathways were PI3K-Akt signaling pathway and MAPK signaling pathway, etc. The GO analysis yielded 1,677 GO entries (*P* < 0.01), such as hormone stimulation, inflammatory response, etc. The top 20 core genes included AKT1, EGFR, IGF1, MAPK1, MAPK10, MAPK14, etc. The result of molecular docking demonstrated that MLIF had the strong binding capability to JNK (MAPK10). The *in vitro* and *in vivo* studies also confirmed that MLIF protected against IS by lowering JNK (MAPK10) and AP-1 levels and decreasing pro-inflammatory cytokines (IL-1, IL-6).

**Conclusion:**

MLIF may exert a cerebral protective effect by inhibiting the inflammatory response through suppressing the JNK/AP-1 signaling pathway.

## Introduction

Stroke is a group of acute and serious cerebral vascular diseases with a high incidence, disability, death, and recurrence rate (1). More than 80% of all conditions result in ischemic stroke (IS), which is triggered by arterial blockage (2). However, the mechanisms involved in the stroke process are complex, such as excitotoxicity, calcium overload, oxidative stress, inflammatory and apoptosis (3, 4). Dyslipidemia is an important risk factor for IS and contributes to IS by several of these mechanisms (5). Elevated blood lipid levels can cause oxidative stress by increasing the production of excess oxygen free radicals, which can alter the arterial and microcirculatory systems (6). Thrombolysis is the most effective treatment for stroke worldwide, although its therapeutic efficacy is greatly controlled by time, and the prognosis is dismal because the majority of patients incur various degrees of neurological impairment after treatment (7). In summary, it is crucial to create effective neuroprotective medications in order to reduce brain damage in stroke patients and improve their prognosis.

Entamoeba histolytica produces a pentapeptide known as monocyte locomotion inhibitory factor (MLIF), which has blatant anti-inflammatory properties (8), such as inhibition of human monocyte motility and immune responses of monocytes and neutrophils, increase of anti-inflammatory cytokines (9), downregulation of LPS-induced IL-1β in U-937 cells (10), and inflammation and matrix metalloproteinases in a collagen-induced arthritis (CIA) mouse model. According to our findings (11), the amount of cerebral ischemia in the temporary middle cerebral artery occlusion (tMCAO) model is greatly decreased by MLIF. Currently, as an experimental novel medication with potential for neuroprotection in acute ischemic stroke, MLIF has been approved (11), but the exact mechanism is unknown.

**Figure 1:**
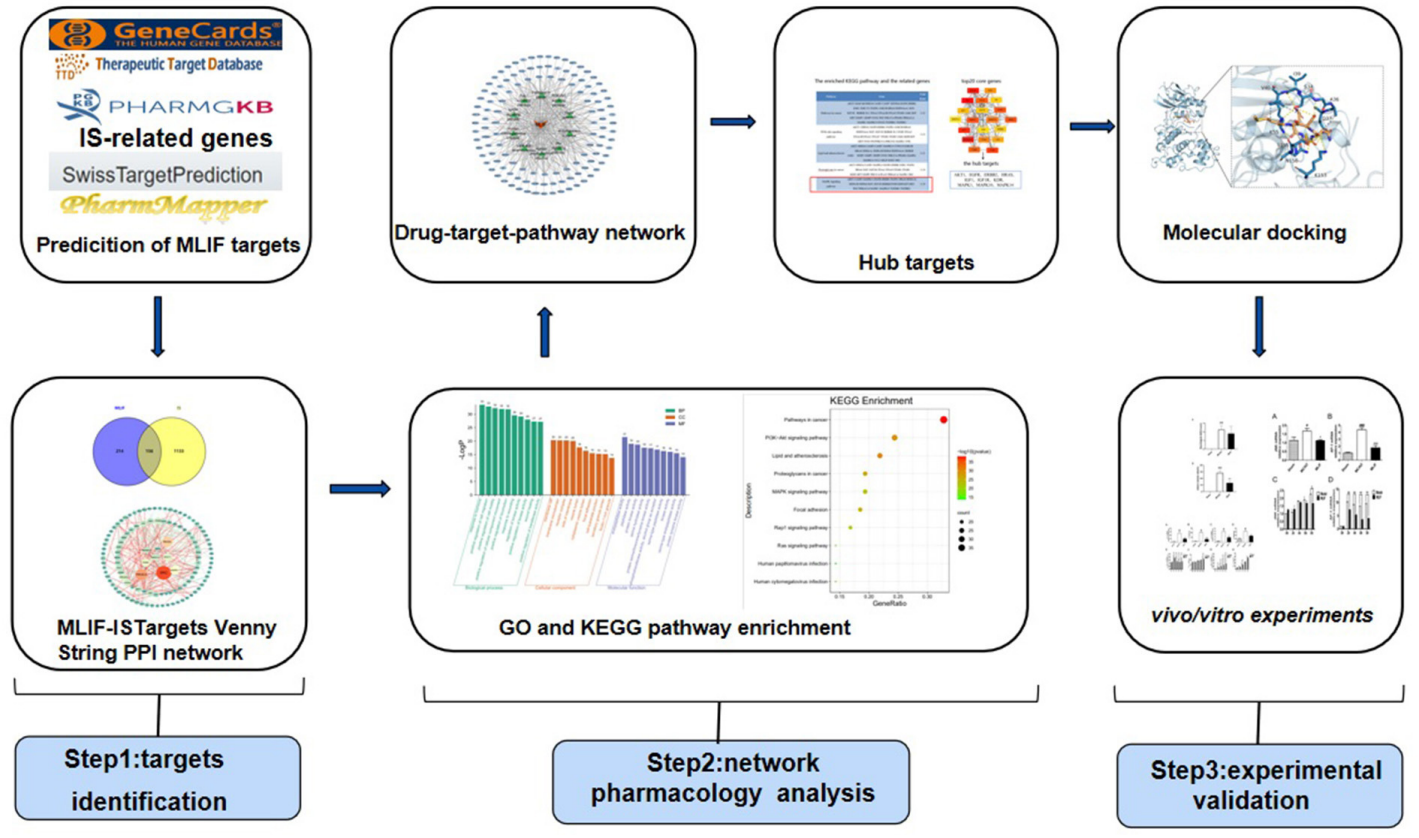
Flowchart of the mechanisms of MLIF against IS.

The network pharmacology approach combines high-throughput data incorporation, metadata search, data analysis, target prediction, and simulation labs to independently present the “drug-target-pathway” associated with a particular disease and to show the connections and interactions between drugs and targets from a systematic perspective (12). It provides a novel tool for innovative drug discovery (13). The process of “molecular docking” involves interacting a drug molecule with a receptor to design new drugs (14), which has become frequently employed in drug discovery (15). Therefore, this study used Swiss TargetPrediction, PharmMapper databases to forecast the MLIF-related targets, and GeneGards, PharmGKB, and Therapeutic Target Database (TTD) to search for targets of IS. After obtaining common targets, the protein-protein interaction (PPI) network, Gene Ontology (GO) functional analysis, and pathway enrichment from the Kyoto Encyclopedia of Genes and Genomes (KEGG) were performed using online analytic platforms like STRING11.0, Metascape, and combined with the cytoHubba plug-in in Cytoscape3.9.1 to extract the top20 core genes. Finally, molecular docking was performed by AutoDock Vina1.1.2. The probable targets of MLIF were further confirmed by tests based on the findings of network pharmacology analysis and molecular docking analysis. From the perspective of network pharmacology, the scientific connotation of the anti-inflammatory effect of MLIF in stroke was elucidated in order to serve as a reference for its use in treating post-stroke neurological damage and to lay the foundation for further studies on its mechanism of action (the flow of the study is shown in Figure 1).

## Materials and methods

### Network pharmacology analysis

#### Prediction of MLIF and IS-related targets

The MLIF structural data was retrieved from the PubChem database (https://pubchem.ncbi.nlm.nih.gov/) (16), and the MLIF-related targets were predicted in PharmMapper (http://www.lilab-ecust.cn/pharmmapper/) and the Swiss TargetPrediction databases (http://www.swisstargetprediction.ch/) (17, 18), respectively. To create a collection of MLIF-related targets, the targets from the two databases were combined and standardized with the Uniprot database (https://www.uniprot.org/) (19). The IS-related targets were looked for in GeneCards (https://www.genecards.org/) (20), PharmGKB (https://www.pharmgkb.org/) (21) and TTD (http://db.idrblab.net/ttd/) (22) databases using “ischemic stroke” and “cerebral ischemia” as the keywords, respectively.

#### Protein interaction network (PPI) and the core genes finding

The intersection of MLIF-related targets and the IS-related targets datasets was obtained by Venny diagram. The STRING11.0 database (https://cn.string-db.org/) was updated with the integrated target data to obtain the protein-protein interaction (PPI) network and the species must be restricted to “Homo sapiens,” with the confidence score of at least 0.9 (23). The PPI network was made visible by using Cytoscape 3.9.1 (https://cytoscape.org/) (24) and topology analysis was performed using the CytoHubba plug-in to obtain the top 20 targets in terms of degree as the core genes (25).

#### Gene function and pathway enrichment analysis

The Metascape database (https://metascape.org/gp/index.html#/main/step1) was used to import the targets of MLIF treatment IS for Kyoto Encyclopedia of Genes and Genomes (KEGG) pathway enrichment and Gene Ontology (GO) functional analysis with *P* < 0.01 (26).

#### Drug-target-pathway network construction

The “MLIF-target-pathway” network was created using the data from the aforementioned procedures and Cytoscape 3.9.1 software (https://cytoscape.org/) (24), allowing us to see how each node interacts with the active substance (MLIF), potential targets, and enriched KEGG pathways.

#### Molecular docking

The 3D structures of target proteins were obtained from the RCSB protein database (https://www.rcsb.org/) (27), and the 2D structure of MLIF was obtained using the PubChem database (https://pubchem.ncbi.nlm.nih.gov/), drawn in 3D using ChemDraw20 (https://www.chemdraw.com.cn/) (28) and converted to pdb file format using OpenBabel 3.1.1 (http://openbabel.org/wiki/Main_Page) (29). MLIF was set as a ligand and the hub targets were set as receptors. In order to delete water molecules and heteroatoms and add charge and hydrogen atoms, the drug and receptor proteins were added to PyMOL 2.4 (https://pymol.org/2/) and AutoDock 4.2.6 (https://autodock.scripps.edu/). Using AutoDock Vina 1.1.2 (https://autodock.scripps.edu/), the produced medication was molecularly docked to the target protein. PyMOL 2.4 was used to visualize the final conformation, which was chosen based on its best binding energy (30).

### Experimental verification

#### Animals and reagents

The rats were kept in a controlled setting with a 12-h cycle of light and dark and were fed a conventional rat diet along with water. The Institutional Animal Care and Use Committee at Shanghai University oversaw all animal studies and surgical operations to ensure that they adhered to international standards for the humane use of experimental animals. Male Sprague-Dawley rats weighing between 250 and 280 g were randomly assigned to one of three groups (*N* = 3–6): the Sham group (Sham), the MCAO group (MCAO), and the MLIF group (MLIF). Five minutes before reperfusion, 1 mg/kg of MLIF was injected into the tail vein for the MLIF group. Both the Sham group and the MCAO group received the identical dosage of normal saline by tail intravenous injection at the same time.

MLIF was created by the Chinese Peptide Company (Hangzhou, China), with a purity level of above 98% and in accordance with the recognized amino acid sequence (Met-Gln-Cys-Asn-Ser). 2,3,5-triphenyl tetrazolium chloride (TTC) was provided by Jiancheng Biotech Co. (Nanjing, China). The peptide was dissolved to its final concentration (50 g/ml) in PBS (pH 7.4) before being employed for cell therapy. Hyclone (Logan, Utah, USA) provided high/low glucose versions of Dulbecco's modified Eagle's medium (DMEM). Fetal bovine serum (FBS) and 0.25% tryptase were purchased from Gibco (Carlsbad, CA, USA). Penicillin-streptomycin solution was purchased from Biosera (France). R&D (Minneapolis, MN, USA) provided the enzyme-linked immunosorbent assay (ELISA) kits for IL-1 and IL-6, and Takara (Shiga, Japan) provided the kits for total RNA. All the other chemicals were bought from businesses.

#### Cell culture

Brain microvascular endothelial cells (bEnd3 cells) were purchased from the American Type Culture Collection (ATCC). The DMEM with high glucose, 10% fetal bovine serum, and 100 g/ml penicillin-streptomycin solution was used to cultivate the bEnd3 cells. At 37°C, cells were grown in 5% CO_2_ and 95% air.

#### Transient middle cerebral artery occlusion

Procedures for tMCAO were previously described (31). In a nutshell, pentobarbitone (350 mg/kg, i.p.) was used to anesthetize rats during surgery. The internal carotid artery was used to insert an 11-mm silicone-coated 8–0 filament into the left common carotid artery for 2 h. The filament was then gently removed to complete a 24-h reperfusion period. A heating lamp was used to keep the subject's body temperature at 37.5 ± 0.5°C. After reperfusion for 24 h, we assessed the efficacy of MLIF by TTC staining and neurological deficit score and measured pro-inflammatory cytokines, including IL-1 and IL-6, in the ischemic penumbra.

#### Oxygen and glucose deprivation

According to a prior description by Yang et al., an ischemic model was created *in vitro* by depriving cells of oxygen and glucose (32). The bEnd3 cells was planted in cell culture plates with 5% CO_2_ at 37°C at a density of 3 × 10^5^ cells/ml. When the confluency reached to 80%, the pure culture was quickly changed to low glucose DMEM without FBS and quinolone solution, The cells were then starved for 2 h in a 95% atmosphere, 5% carbon dioxide atmosphere at 37°C. The OGD insult was administered after MLIF (50 g/ml) administration, and the cells were placed under hypoxic conditions (5% CO_2_ and 95% N_2_) for various time periods (0, 2, 4, 6, and 8 h) in different experiments. The ELISA assay and Quantitative real-time PCR (qRT-PCR) were performed after OGD to detect the inflammatory cytokines (IL-1 and IL-6).

#### Evaluation of neurological deficit score

We used Longa's approach to calculate the neurological deficit score for rats (0 = no neurological deficit, 1 = unable to extend left front paw, 2 = rats' crawling motion circled to the left, 3 = rats stood with a left-leaning posture, 4 = rats lost consciousness and were unable to walk on its own) (33).

#### Staining with 2,3,5-triphenyl-tetrazolium chloride

Each group's brains were taken out and preserved for 15 min at −20°C. The brains were divided into six sections, each about 2 mm thick. The sections were immersed in 1% 2,3,5-triphenyl-tetrazolium chloride (TTC) for 30 min at 37°C. Sections were rotated every 5 min and then washed three times with ddH2O. Images were gathered for additional examination.

#### Enzyme-linked immunosorbent assay

According to the instructions provided by the kit's manufacturer (R&D), the ELISA was used to measure the amounts of inflammatory cytokines (IL-1 and IL-6) in the hydrolysates of ischemic brain tissue and the effluent of the bEnd3 cells. The reactions were placed in ELISA plates, and the results were examined at a wave length of 450 nm. The standard curve was used to determine the concentrations of IL-1 and IL-6.

#### Quantitative real-time PCR

To evaluate the RNA levels of IL-1, IL-6, JNK, and AP-1, qRT-PCR was utilized. Total RNA was extracted from brain tissues and bEnd3 cells using the Total RNA Kit (Takara, Shiga, Japan). According to the manufacturer's instructions, cDNAs were created using 5 × Primescript reverse transcription reagents (Takara, Shiga, Japan). Table 1 displayed the primers being used in quantitative RT-PCR (qRT-PCR). qRT-PCR was carried out using SYBR Premix Ex Taq^TM^ (Tli RnaseH Plus) (Takara, Shiga, Japan) on 7500 Real-Time PCR System (Applied Biosystems). The level of expression for each target genes was normalized to the control (GAPDH).

**Table 1:** Sequence of the qRT-PCR primers.

**Gene**	**Sequence of primers**
Mouse IL-1	F CAACCAACAAGTGATATTCTCCATG R GATCCACACTCTCCAGCTGCA
Mouse IL-6	F TCCAGTTGCCTTCTTGGGAC R GTGTAATTAAGCCTCCGACTTG
Mouse AP-1	F TGGTGGCGTTCGTTTC R CACTGACTTGCTCTTCCCTC
Mouse JNK	F AGTGACAGTAAAAGCGATGG R TTTAGGAGGACAAGTTCACG
Rat IL-1	F AGGCTGACAGACCCCAAAAG R CTCCACGGGCAAGACATAGG
Rat IL-6	F CAGCCACTGCCTTCCCTACTTC R TAGCCACTCCTTCTGTGACTCTAACT
Rat AP-1	F GACTGCAAAGATGGAAACGACC R AGAAGGTCCGAGTTCTTGGC
Rat JNK	F CCAAGAGAGCTTATCGGGAAC R TCCCAAGATGACTTCTGGAGC
GAPDH	F ACCACAGTCCATGCCATCAC R TCCACCACCCTGTTGCTGTA

#### Statistical analysis

Every piece of data was reported as mean±SD. Using one-way analysis of variance (ANOVA), statistical analysis was carried out. The Student's *t*-test was applied to compare the two groups. Statistics were judged significant at *P* < 0.05. With GraphPad Prism 9.0 (SPSS Inc., United States), all statistical analysis was completed.

## Results

### Prediction of drug and disease targets

**Figure 2:**
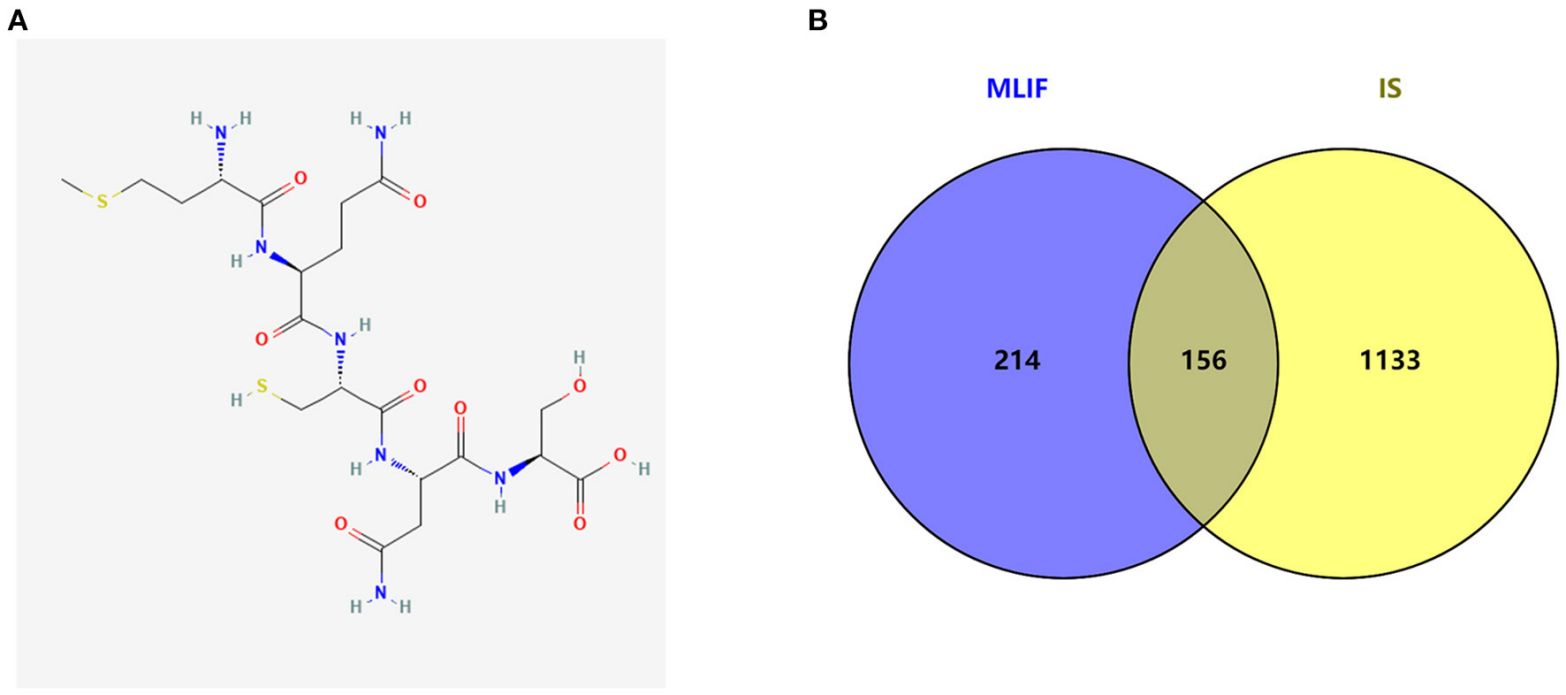
The graph structure of MLIF **(A)** and target intersections for MLIF and IS **(B)**.

The MLIF 2D structure from the pubchem database (CID: 10325914 Figure 2A), MLIF-related targets prediction was performed using the Swiss TargetPrediction platform and PharmMapper databases, and 370 action objectives in total were found after de-duplication screening (Supplementary Table 1). After deleting entries with duplicate information from the GeneCards, PharmGKB, and TTD databases, 1,289 targets associated to IS were found.

### Construction of PPI network

**Figure 3:**
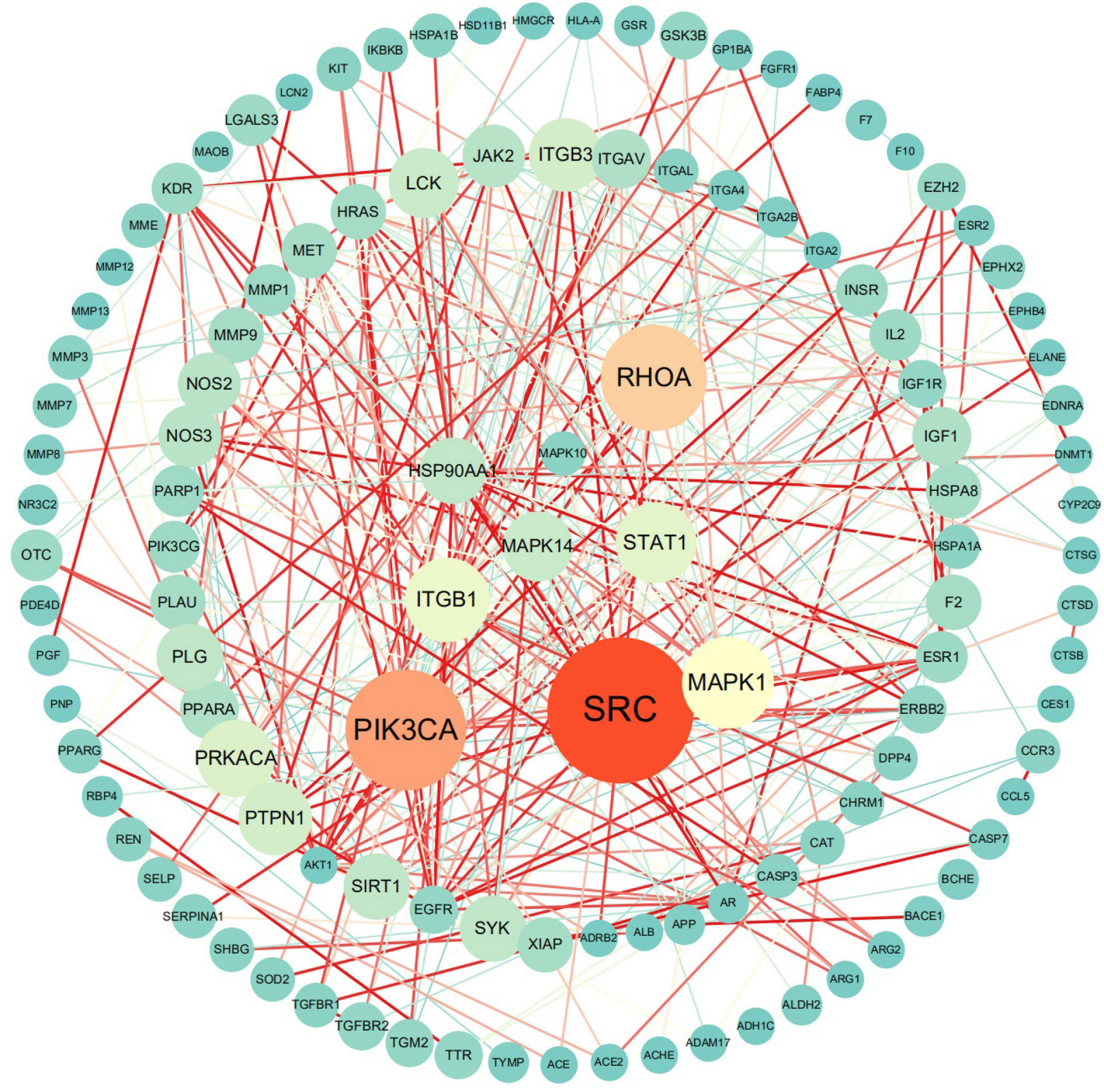
PPI network, where nodes stand in for proteins and edges for connections.

**Figure 4:**
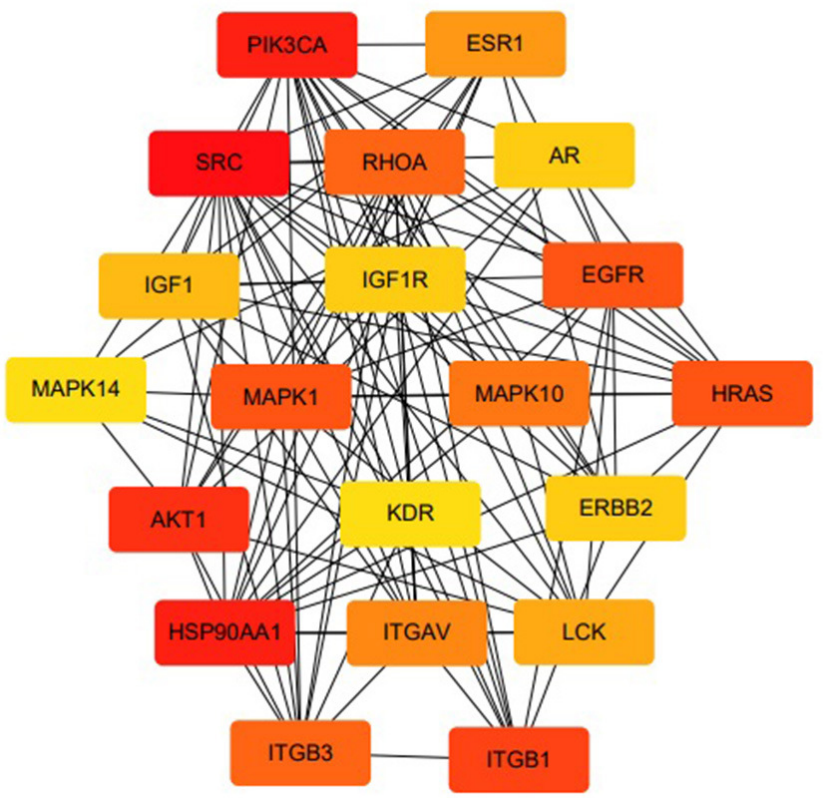
Top 20 core genes in terms of degree.

The MLIF-related targets and IS-related targets were intersected to obtain 156 common targets between drug and disease (Figure 2B). The 156 common targets were imported into the STRING11.0 database, and targets with the confidence score > 0.9 were screened out, and isolated targets were excluded to obtain a PPI network containing 119 targets. It is suggested that these 119 shared targets may be candidate targets for MLIF treatment of IS. The PPI network was shown in Figure 3, where each edge represented a protein interaction and each node represented a target. The size of the nodes indicates the degree's magnitude. The network diagram demonstrates the intimate ties between the targets, demonstrating the complexity and diversity of the MLIF for the treatment of IS and the synergy between various targets. The top 20 core genes were determined using Cytoscape 3.9.1's cytoHubba plug-in, and they were shown in Figure 4. The node color and score are correlated, with darker and more red nodes signifying higher scores.

### GO and KEGG enrichment analysis of candidate targets

**Figure 5:**
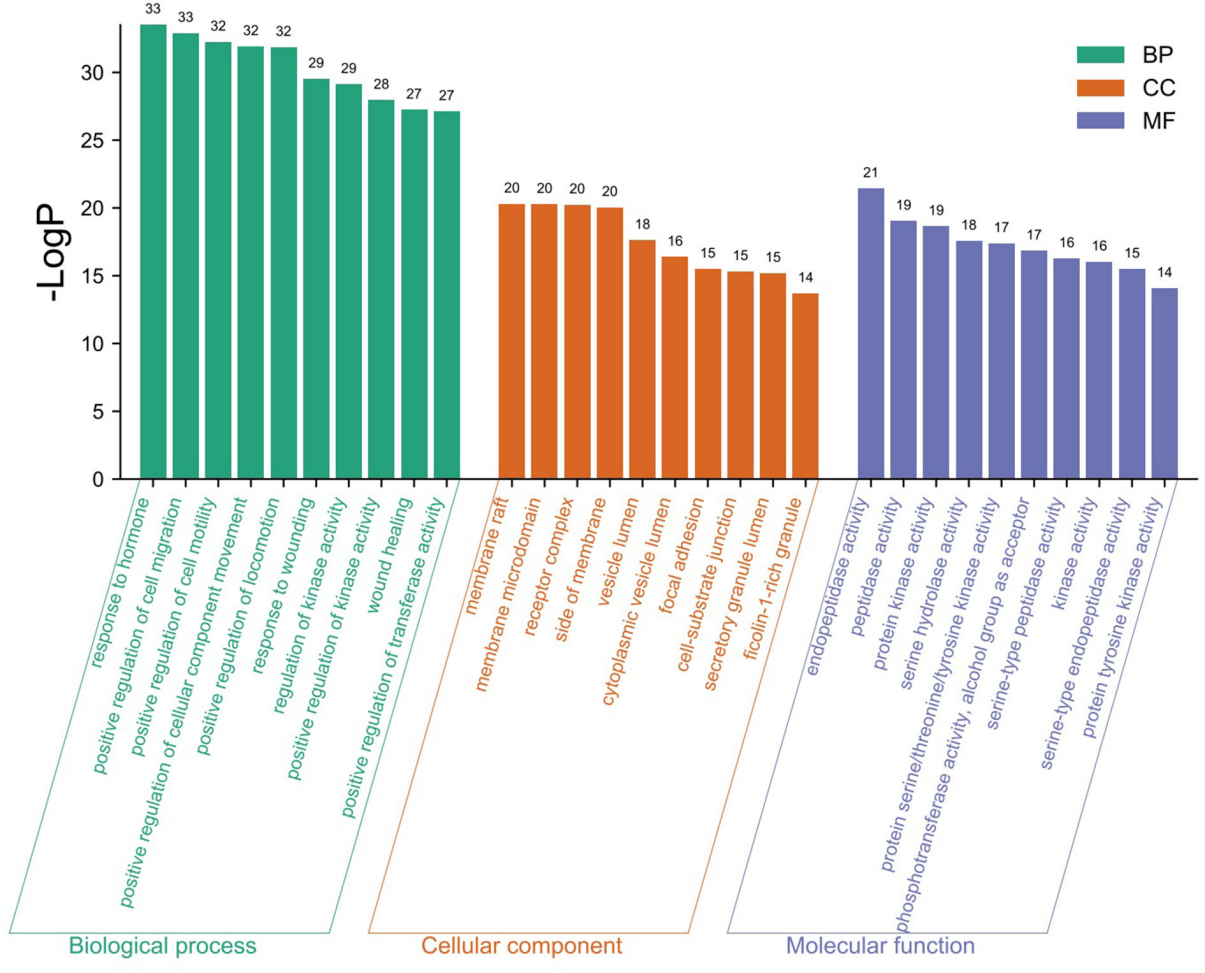
GO enrichment analysis findings.

**Figure 6:**
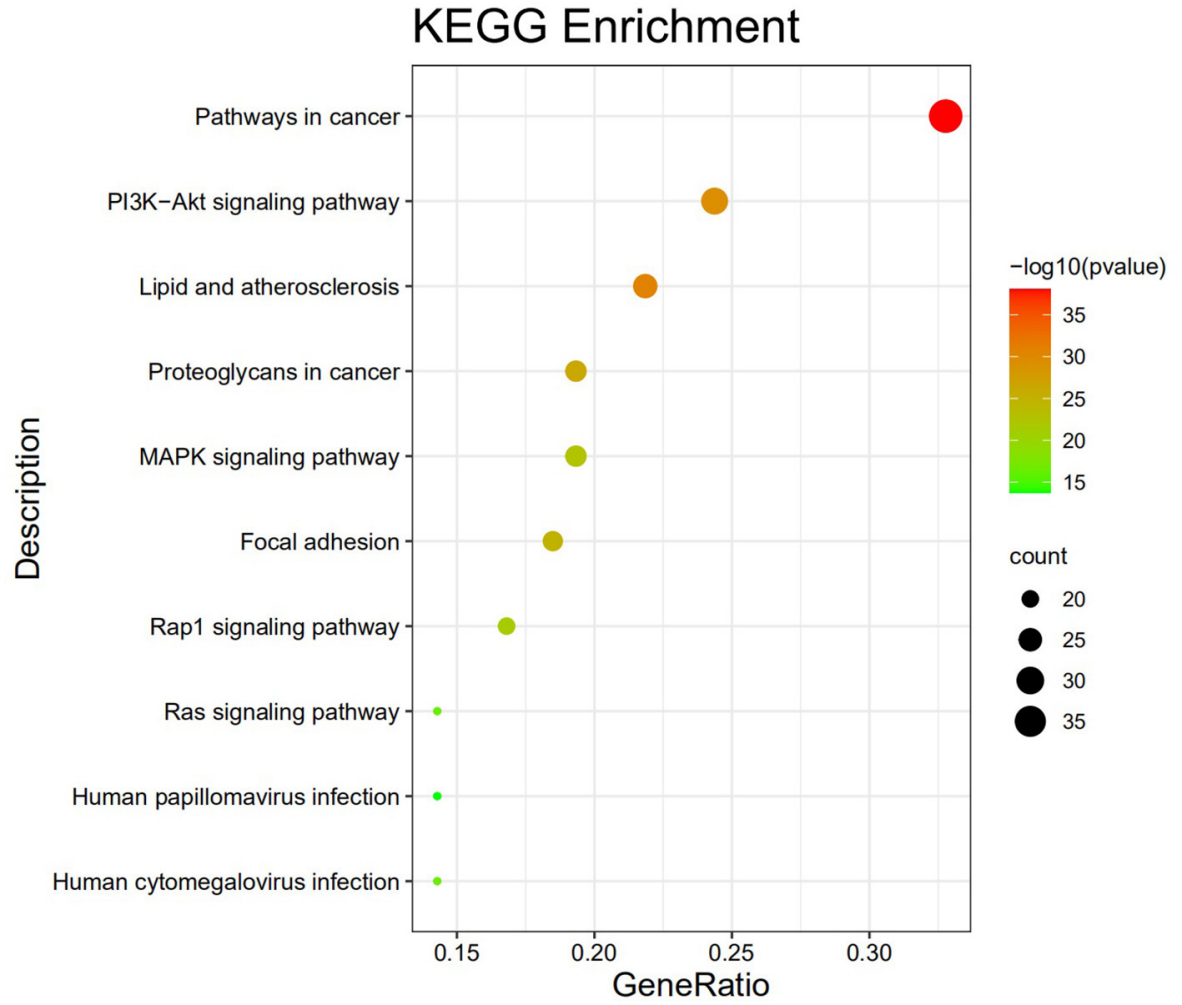
Top ten KEGG pathway enrichment findings.

**Figure 7:**
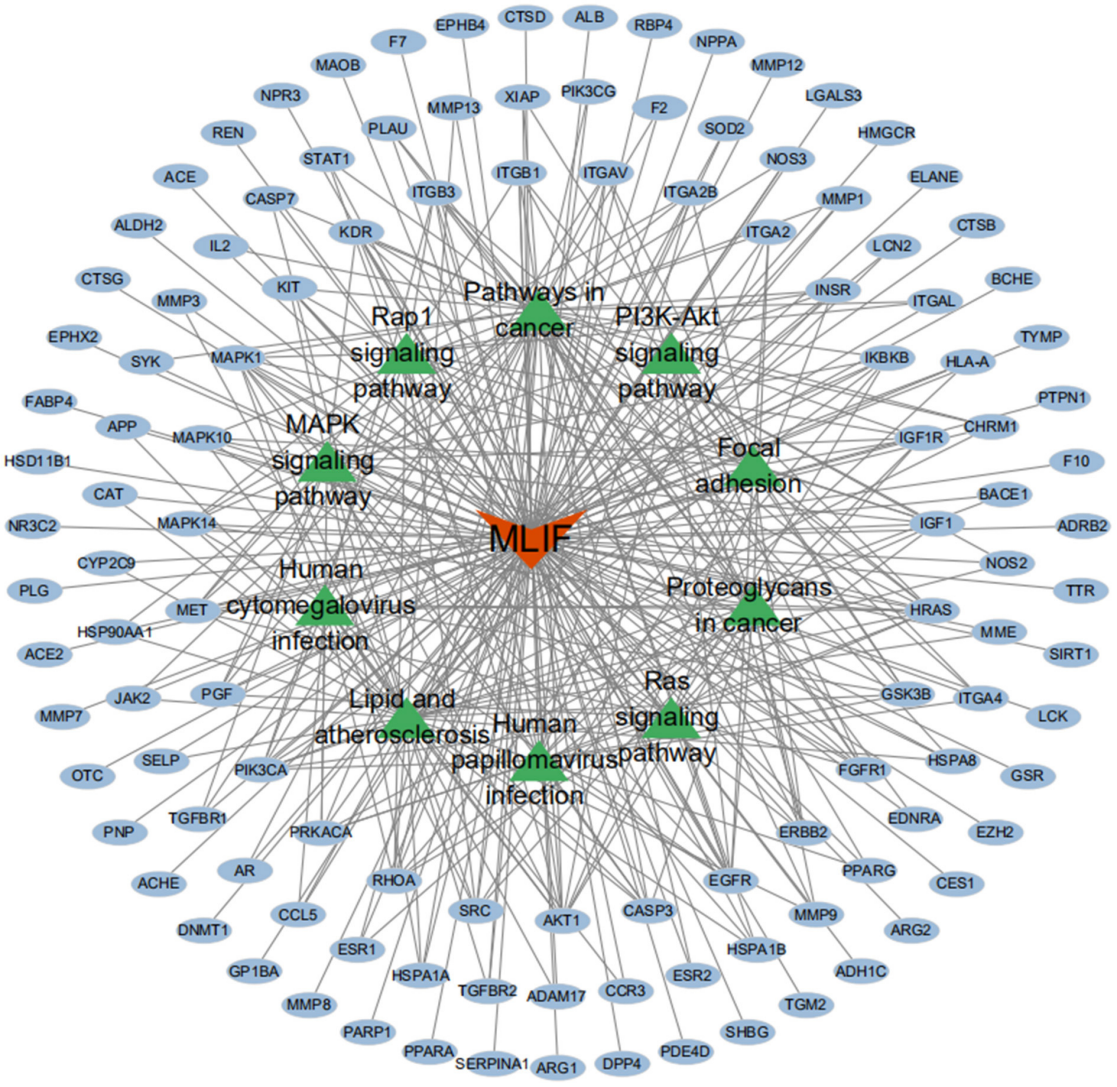
MLIF-target-pathway network. MLIF, targets, and KEGG pathways are represented by the red V shape, blue circles, and green triangle circles, respectively.

The GO analysis by Metascape database yielded 1,677 GO entries (*P* < 0.01), including 1,437 entries for biological process (BP), such as positive regulation of cell migration, hormone stimulation, positive regulation of cellular component movement, etc, 97 entries for cellular component (CC), such as cell membrane, cytoplasm, cytosol, nucleus, etc., 143 entries for molecular function (MF), such as endopeptidase activity, peptidase activity, protein kinase activity, etc. For visualization, the best 10 submissions for each category were selected and shown in Figure 5. The KEGG pathway enrichment of 119 candidate targets yielded 181 signaling pathways with *P* < 0.01, mainly involving neural, vascular, inflammation, oxidative stress, apoptosis and lipid metabolism. Ten pathways with more significant gene ratio in the enrichment results were selected for display (Figure 6), those related to other diseases including cancer signaling pathways, human papillomavirus infection, human cytomegalovirus infection, lipids and atherosclerosis. And those related to apoptosis: PI3K-Akt signaling pathway, RAS signaling pathway, two related to adhesion: Rap1 signaling pathways and Focal adhesion. The inflammation-related pathways: PI3K-Akt signaling pathway and mitogen-activated protein kinase (MAPK) signaling pathway. A total of 23 targets were involved in the MAPK signaling pathway (Table 2). The “MLIF -target-pathway” network was further built using nodes of various colors and shapes to represent various active compound, targets, and pathways. One compound (red V-shape), 119 targets (blue circles), and 10 KEGG pathways (green triangles) are among the nodes that represent active compounds, targets, and pathways (Figure 7).

**Table 2:** The top 10 KEGG pathways that are enriched and the associated genes.

**Pathway**	**Gene**	**Gene ratio**
Pathways in cancer	AKT1 XIAP AR RHOA CASP3 CASP7 EDNRA EGFR ERBB2 ESR1 ESR2 F2 FGFR1 GSK3B HRAS HSP90AA1 IGF1 IGF1R IKBKB IL2 ITGA2 ITGA2B ITGAV ITGB1 JAK2 KIT MET MMP1 MMP9 NOS2 PGF PIK3CA PPARG PRKACA MAPK1 MAPK10 STAT1 TGFBR1 TGFBR2	0.32
PI3K-Akt signaling pathway	AKT1 CHRM1 EGFR ERBB2 FGFR1 GSK3B HRAS HSP90AA1 IGF1 IGF1R IKBKB IL2 INSR ITGA2 ITGA2B ITGA4 ITGAV ITGB1 ITGB3 JAK2 KDR KIT MET NOS3 PGF PIK3CA PIK3CG MAPK1 SYK	0.24
Lipid and atherosclerosis	AKT1 RHOA CASP3 CASP7 MAPK14 CYP2C9 GSK3B HRAS HSPA1A HSPA1B HSPA8 HSP90AA1 IKBKB JAK2 MMP1 MMP3 MMP9 NOS3 PIK3CA PPARG MAPK1 MAPK10 CCL5 SELP SOD2 SRC	0.21
Proteoglycans in cancer	AKT1 RHOA CASP3 MAPK14 EGFR ERBB2 ESR1 FGFR1 HRAS IGF1 IGF1R ITGA2 ITGAV ITGB1 ITGB3 KDR MET MMP9 PIK3CA PLAU PRKACA MAPK1 SRC	0.19
MAPK signaling pathway	AKT1 CASP3 MAPK14 EGFR ERBB2 FGFR1 HRAS HSPA1A HSPA1B HSPA8 IGF1 IGF1R IKBKB INSR KDR KIT MET PGF PRKACA MAPK1 MAPK10 TGFBR1 TGFBR2	0.19
Focal adhesion	AKT1 XIAP RHOA EGFR ERBB2 GSK3B HRAS IGF1 IGF1R ITGA2 ITGA2B ITGA4 ITGAV ITGB1 ITGB3 KDR MET PGF PIK3CA MAPK1 MAPK10 SRC	0.18
Rap1 signaling pathway	AKT1 RHOA MAPK14 EGFR FGFR1 HRAS IGF1 IGF1R INSR ITGA2B ITGAL ITGB1 ITGB3 KDR KIT MET PGF PIK3CA MAPK1 SRC	0.17
Ras signaling pathway	AKT1 RHOA EGFR FGFR1 HRAS IGF1 IGF1R IKBKB INSR KDR KIT MET PGF PIK3CA PRKACA MAPK1 MAPK10	0.14
Human cytomegalovirus infection	AKT1 RHOA CASP3 CCR3 MAPK14 EGFR GSK3B HLAA HRAS IKBKB ITGAV ITGB3 PIK3CA PRKACA MAPK1 CCL5 SRC	0.14
Human papillomavirus infection	AKT1 CASP3 EGFR GSK3B HLAA HRAS IKBKB ITGA2 ITGA2B ITGA4 ITGAV ITGB1 ITGB3 PIK3CA PRKACA MAPK1 STAT1	0.14

### Molecular docking results

**Figure 8:**
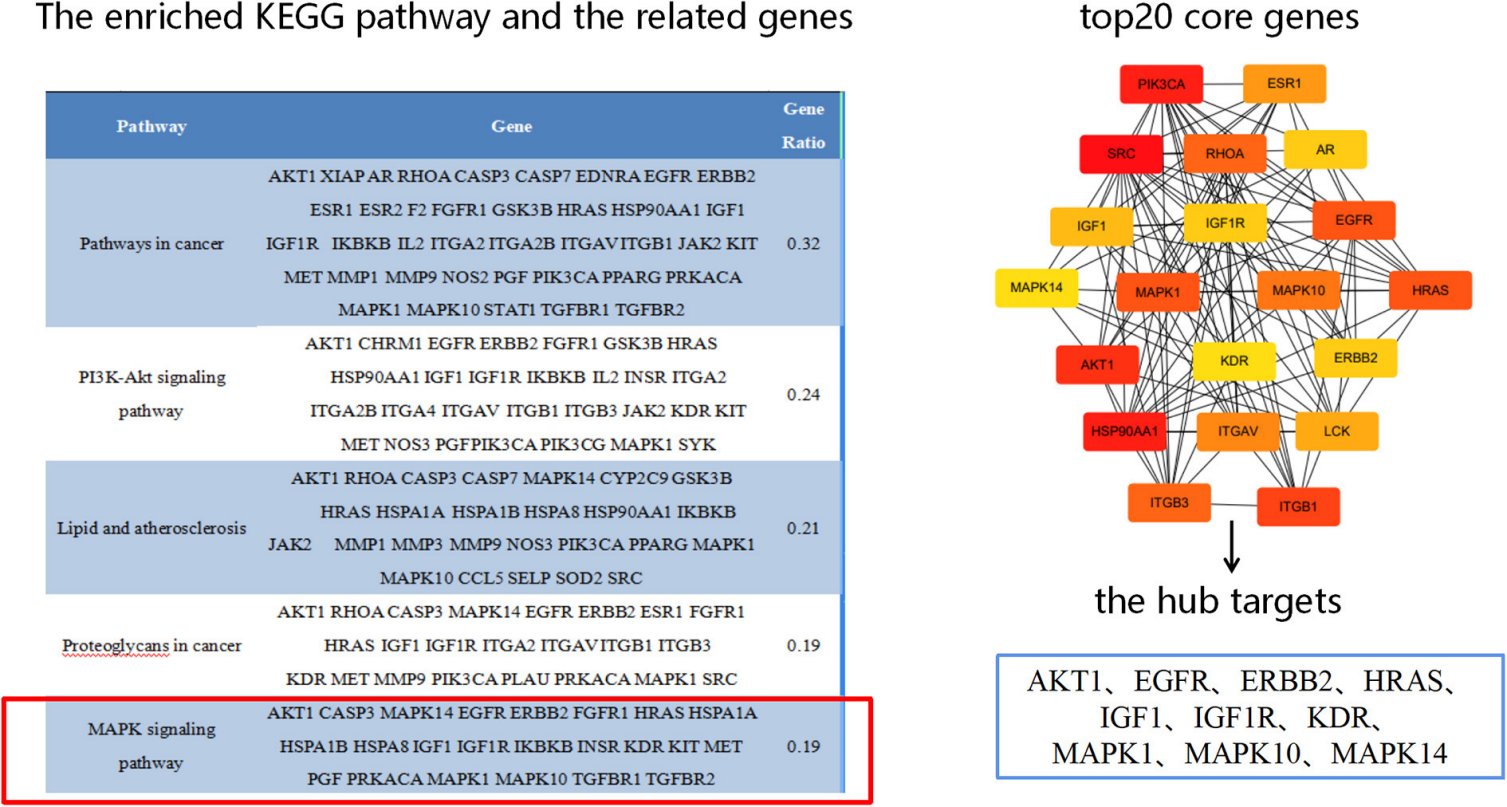
The hub targets.

**Figure 9:**
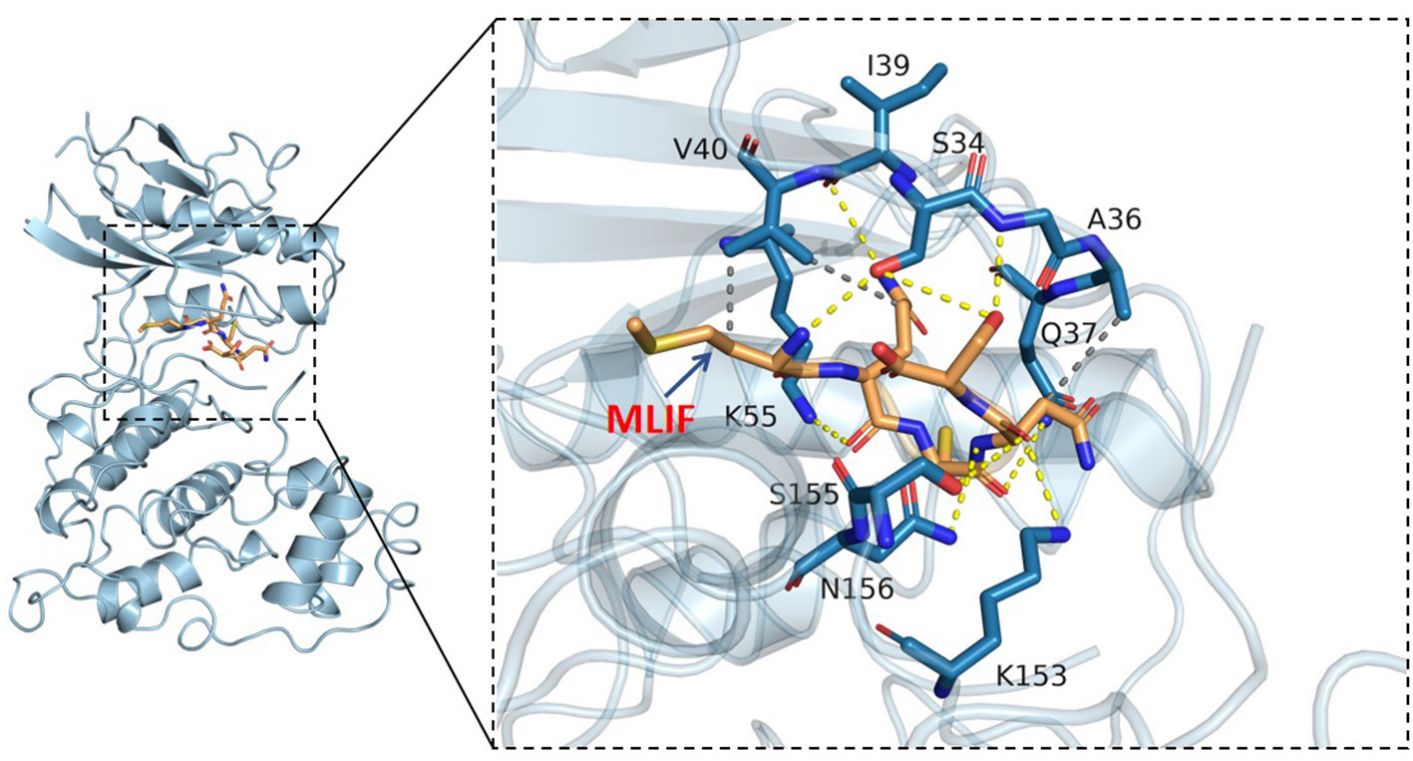
3D docking conformation of MLIF with JNK (MAPK10). The **orange** stick in the diagram is a small molecule (MLIF), the **blue** cartoon is a protein (JNK), the **yellow** dashed line indicates hydrogen bonding and the **gray** dashed line indicates hydrophobic interactions.

Based on the results of KEGG analysis and top20 core genes analysis, AKT1 (PDB ID: 6s9w), EGFR (PDB ID:5Y9T), ERBB2 (PDB ID:2a91), HRAS (PDB ID:5P21), IGF1 (PDB ID:1b9g), IGF1R (PDB ID:6PYH), KDR/VEGFR2 (PDB ID:1y6a) MAPK1/ERK (PDB ID:6G54), MAPK10/JNK (PDB ID:7S1N), MAPK14/P38 (PDB ID:5ETI) were selected as the hub targets (Figure 8). As shown in Table 3, MLIF was selected as the ligand and the hub targets were selected as receptors. There's a strong possibility that MLIF binds well to the JNK receptor (≤ -7.0 kcal/mol) (Figure 9), a protein corresponding to MAPK10, and this indicates that MLIF has good binding potential to key targets of the JNK signaling pathway.

**Table 3:** The docking information of hub targets with MLIF.

**Gene**	**Binding energy (kcal/mol)**
MAPK10	−7.2
EGFR	−5.9
ERBB2	−6.3
IGF1	−4.5
IGF1R	−5.6
KDR	−5.7
AKT1	−3.9
HRAS	−2.7
MAPK1	−1.5
MAPK14	−0.5

### MLIF played a protective role against neurological damage on the MCAO rats

**Figure 10:**
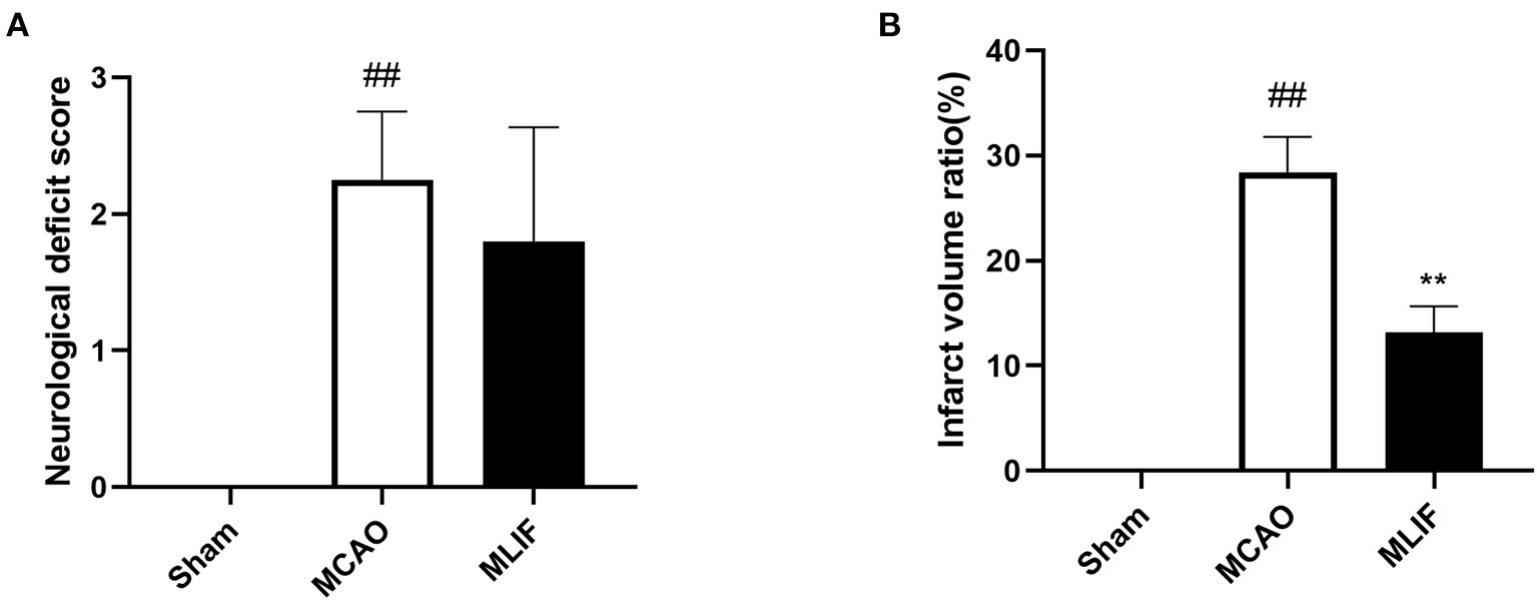
MLIF played a protective role against neurological damage on the MCAO rats. Neurological deficit score **(A)** and infarct volume statistical results **(B)** of brain tissue in rats. (*N* = 6, ^##^*P* < 0.01 vs. Sham group, **P < 0.01 vs. MCAO group).

The neurological deficit score of the MCAO group was considerably higher than that of the Sham group, as can be seen in Figure 10A (*P* < 0.01). Although the MLIF group's neurological deficit score was lower than the MCAO group's, there was no discernible difference between the two. The focal infarct volume was also measured 24 h following reperfusion. As shown in Figure 10B, the percentage of infarct volume in the MCAO group was considerably higher than that in the Sham group (*P* < 0.01), and MLIF significantly decreased the percentage of infarct volume in the MCAO group (*P* < 0.01).

### MLIF reduced the expression of the IL-1 and IL-6 in the MCAO rat's brain and OGD insulted bEnd3 cells

**Figure 11:**
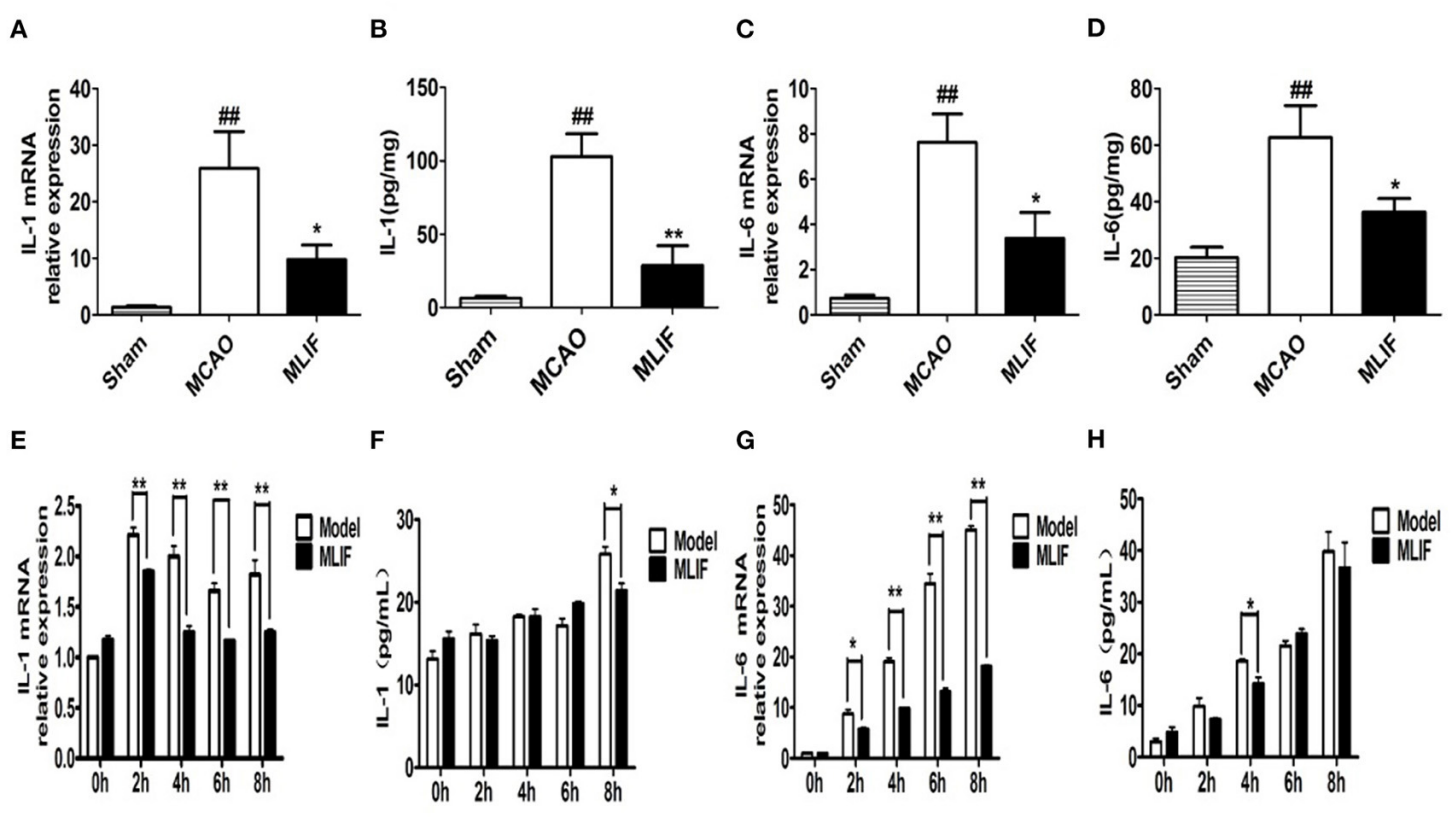
MLIF reduced the expression of the pro-inflammatory cytokines IL-1 and IL-6 in the MCAO rats and bEnd3 cells at the different time after OGD. The relative mRNA expression levels of IL-1 **(A,E)** and IL-6 **(C,G)** in the MCAO rats and bEnd3 cells were determined by qRT-PCR. IL-1 **(B,F)** and IL-6 **(D,H)** in the rat brain tissue homogenate and OGD-insulted bEnd3 cells supernatant was measured by ELISA assay (*N* = 3, ^##^*P* < 0.01 vs. Sham group, **P < 0.01 vs. MCAO group, *P < 0.05 vs. MCAO group).

The expansion of the area with brain injury brought on by the release of pro-inflammatory cytokines into the extracellular space raises the neurological deficit and has a detrimental effect on the prognosis for survival (34). IL-1 receptor antagonist (IL**-**1Ra) administration significantly reduced peripheral markers of inflammation in patients with ischemic stroke (35, 36). We determined the effect of MLIF on cytokines which promote inflammation (IL-1 and IL-6) expression in the pathological process of brain ischemia by qRT-PCR and ELISA analysis *in vivo* and *in vitro*, respectively. We found that MLIF (1 mg/kg) could reduce the relative IL-1 and IL-6 mRNA expression (*P* < 0.05) in the MCAO rats (Figures 11A,B). It also showed a significant reduction of IL-1 (*P* < 0.01) and IL-6 (*P* < 0.05) in the MCAO rats (Figures 11C,D). In this *in vitro* study, we used the OGD model and found that the relative mRNA expression of IL-1 and IL-6 were significantly upregulated at the different time (2, 4, 6, and 8 h) after OGD (Figures 11E,G). Administration of MLIF (50 μg/ml) remarkably reduced the expression of IL-1 compared with the OGD model group (2, 4, 6, and 8 h) (*P* < 0.01). As for IL-6, MLIF has the similar effect at 2 h (*P* < 0.05),4 h, 6 h, and 8 h (*P* < 0.01) after OGD insult. Moreover, we also assessed protein expression of IL-1 and IL-6 in bEnd3 cells supernatant after OGD by ELISA. As shown in Figures 11F,H, MLIF significantly decreased the level of IL-1 (*P* < 0.05) at 8 h and IL-6 at 4 h (*P* < 0.05) in bEnd3 cells' supernatant after OGD insult. These results showed the anti-inflammatory effect of MLIF on brain ischemia.

### MLIF inhibited the JNK/AP-1 signaling pathway in the MCAO rats and OGD insulted bEnd3 cells

**Figure 12:**
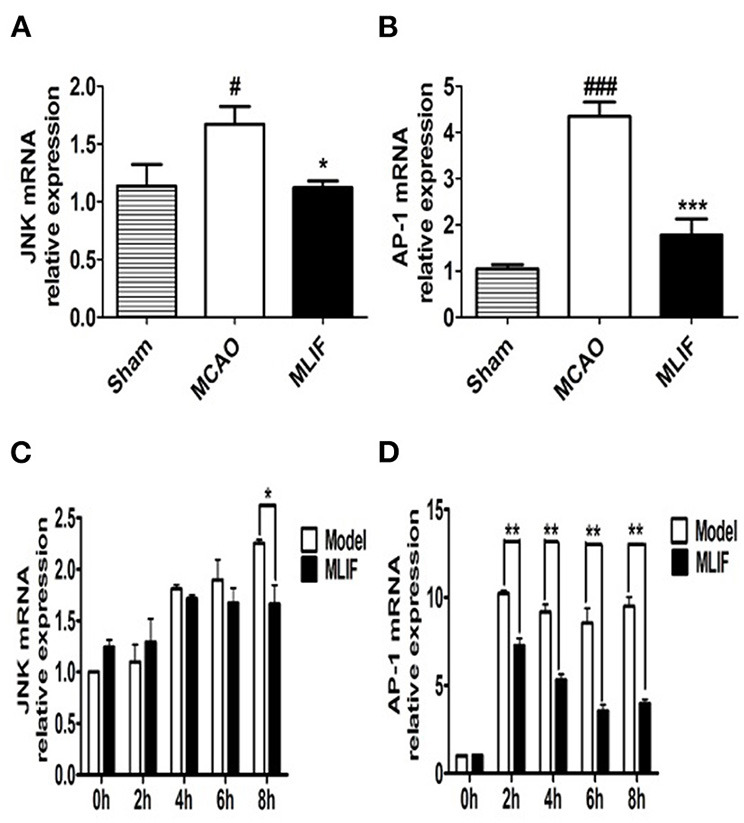
JNK/AP-1 signaling pathways contributes to the anti-inflammatory effect of MLIF. The relative mRNA levels of JNK **(A,C)** and AP-1 **(B,D)** in rat's brain and bEnd3 cells were determined by qRT-PCR at the different time (0, 2, 4, 6, and 8 h) after OGD (*N* = 3, ^###^*P* < 0.001 vs. Sham group, ^#^*P* < 0.05 vs. Sham group, ***P < 0.001 vs. MCAO group, **P < 0.01 vs. MCAO group, *P < 0.05 vs. MCAO group).

Studies have suggested that the JNK pathway's most significant downstream, AP-1 activation, may have a role in the expression of numerous genes linked to cell survival or death in ischemic brain injury (37). So we further measured JNK, AP-1 levels in the rats brain tissue after MCAO and bEnd3 cells during the OGD insult. MLIF (1 mg/kg) could reduce the relative mRNA expression of JNK (*P* < 0.05) (Figure 12A) and AP-1 (*P* < 0.001) (Figure 12B) *in vivo*. Treatment of MLIF (50 μg/ml) resulted in a significant reduction in the transcriptional level of JNK in bEnd3 cells after OGD insult when it was at 8 h (*P* < 0.05) (Figure 12C). And MLIF (50 μg/ml) could remarkably decreased OGD-induced the gene expression of AP-1 at 2, 4, 6, and 8 h (*P* < 0.01) (Figure 12D). These findings suggested that MLIF may inhibit inflammatory responses after IS *in vivo* and *in vitro* through the JNK/AP-1 signaling pathways.

## Discussion

According to some researchers, the heightened inflammatory response may have a role in the development of stroke (38). In general, inflammatory indicators like IL-6 produce cardiovascular remodeling and arterial resistance (39) and increase the incidence of cardiovascular disease (CVD) (40).

Entamoeba Histolytica produces the heat-stable pentapeptide MLIF, which has a variety of anti-inflammatory effects (9, 41, 42), including regulating inflammation and immune responses through the NF-κB and MAPK pathways (43). The anti-inflammatory effect of MLIF makes the its increasing research on brain ischemia (44). Our previous study has proved that MLIF can reduce the risk of brain ischemia injury by focusing on the eEF1A1/eNOS pathway (45).

In recent years, network pharmacology has become frequently employed to forecast novel drug targets and pathways, lessening the blindness of research and improving the efficiency of drug discovery (46). Molecular docking techniques have been used to validate the predictions of network pharmacology by combining components with target proteins in a virtual evaluation (47). The network pharmacology was used in the study to further analyze the possible targets and pathways of MLIF for the treatment of IS and found that MLIF acts on top20 core genes, includingAKT1, EGFR, ERBB2, HRAS, IGF1, MAPK1, MAPK10, MAPK14, etc. The “MLIF-target-pathway” network analysis revealed that the effect of MLIF on IS therapy PI3K/AKT, MAPK, and other signaling pathways were involved. PI3K/AKT signaling pathway is a pro-cell survival pathway that regulates cell proliferation, differentiation, metabolism, and anti-apoptosis (48). This pathway is activated in the early stages of cerebral ischemia, inducing apoptosis and inflammatory response, and its activity is gradually inhibited as ischemia increases (49). The MAPK signaling pathway is crucial for controlling inflammation, neuronal apoptosis, brain edema, and ischemia-reperfusion injury. It also affects the development of stroke (50). Stress-activated protein kinase (SAPK) or Jun amino-terminal kinase (JNK) is a member of the MAPK family and is activated by a range of environmental stresses, inflammatory cytokines, and growth factors. It is crucial for stress reactions like apoptosis and inflammation (51). The JNK pathway is thought to be a marker of neuronal cell death. JNK phosphorylation can result in the transcription of inflammatory mediators like nitric oxide (NO), inducible nitric oxide synthase (iNOS), cyclooxygenase-2 (COX-2), prostaglandin E2 (PGE2), and the pro-inflammatory cytokines IL-6 and IL-1, which in turn can result in neuronal degeneration.

Based on the results of the network pharmacology, the study was conducted to validate the hub targets of the MAPK signaling pathway using molecular docking techniques and *vivo/vitro* experiments, respectively. And MLIF showed strong binding capacity ( ≤ -7.0 kcal/mol) to JNK (MAPK10). In addition, *in vitro* and *in vivo* experiments demonstrated that JNK and AP-1 RNA levels were considerably decreased in the MLIF group, and the transcript and expression of inflammatory factors (IL-1, IL-6) were reduced, further suggesting that MLIF has a therapeutic effect on IS inflammation, possibly by regulating the JNK/AP-1 signaling pathway (52).

## Data availability statement

The datasets presented in this study can be found in online repositories. The names of the repository/repositories and accession number(s) can be found in the article/Supplementary material.

## Ethics statement

The animal study was reviewed and approved by the Shanghai University Ethics Committee.

## Author contributions

The tests were designed by YZ and JM. Data analysis, figure creation, and manuscript writing were all done by ML. The animal model was built by ML, QZ, XL, SD, and YG. The Western blot and cell tests were carried out by ML. The article's submission was reviewed and approved by all authors.

## Funding

This research was made possible thanks to grants from the Shanghai University School of Medicine's Innovation Fund for Interdisciplinary New Medical Graduate Students and the National Natural Science Foundation of China (No. 81971017).

## Conflict of interest

The authors declare that the research was conducted in the absence of any commercial or financial relationships that could be construed as a potential conflict of interest.

## Publisher's note

All claims expressed in this article are solely those of the authors and do not necessarily represent those of their affiliated organizations, or those of the publisher, the editors and the reviewers. Any product that may be evaluated in this article, or claim that may be made by its manufacturer, is not guaranteed or endorsed by the publisher.
